# News and Events of the Week

**Published:** 1911-07-01

**Authors:** 


### NEWS AND EVENTS OF THE WEEK.

THE Lord Mayor has received £1,000 from Mr. William Herring for the Hospital Sunday Fund.

THE Institute of Puericulture, started by the Conseil général de la Seine, was formally opened on June 8 at the hospice des Enfants-Assistés. Dr. Variot, to whose initiative Paris owes this new foundation, gave an outline of the programme which the Institute was to carry out, and Professor Lucas-Championnière spoke with regard to the important results to be obtained by properly considered instruction.

THE University of Ghent, which possesses an Institute of Physiotherapy furnished with an up-to-date installation, has up to now only issued diplomas of "licentiate" in this science. A recent Royal decree has now created the grade of doctor in physiotherapy for students and doctors of medicine. The courses of instruction are in the hands of Dr. de Nobele, professor of radiology, electro-therapy, and hydrotherapy, and of Dr. Gommaerts, professor of massage and kinesitherapy.

MISS M. A. R. TUKER has received from Her Majesty's Lady-in-Waiting a letter in which she says: "Her Majesty will certainly have much pleasure in accepting the book she kindly offers her on Christian and Ecclesiastical Rome—with the account of the Order of St. John of Jerusalem." The volume containing the account of the ancient Order of St. John of Jerusalem (known on the Continent as the Order of Malta) has now been presented to Her Majesty the Queen, and a copy has also been laid on the author's behalf before the King. Miss Tuker is a Lady-of-Justice of the Order.

THE current edition of the "A B C Guide to the Highlands of Scotland" has just been issued by the Highland Railway. The feature of the guide is an exhaustive description of the routes traversed by the Highland Railway from Perth in the south to John o'Groats in the north and the island of Skye in the west. An original feature of the guide is a wonderfully replete gazetteer arranged alphabetically, embracing all places of interest in the Highlands. Much other valuable information is given for the convenience of tourists, sportsmen, and golfers, including a list of open fishings and golf-courses with descriptive notes. The guide may be had post free on application to the Highland Railway, Head Office, Inverness; 32 Renfield Street, Glasgow; 134 Princes Street, Edinburgh; or to W. T. Hedges, Ltd., Effingham House, Arundel Street, Strand, London, W.C.

ACCORDING to the report of the Chief Inspector of Factories and Workshops, which has recently been issued, it appears that an appreciable diminution in cases of industrial poisoning took place during 1910. In some of the trades in which lead poisoning is liable to occur there has been a sensible decrease, but in the cases of letter-press printing and enamelling an increase is noted as compared with the previous year. Seven cases of arsenical poisoning are reported, none being fatal, and ten cases of mercurial poisoning, with one death. The report of a death from the inhalation of sulphuretted hydrogen is given in detail, and is interesting to students of toxicology on account of the rarity with which this gas has caused fatalities.

